# Short-Term Compound Training on Physical Performance in Young Soccer Players

**DOI:** 10.3390/sports8080108

**Published:** 2020-07-30

**Authors:** Athos Trecroci, Marco Duca, Damiano Formenti, Giampietro Alberti, F. Marcello Iaia, Stefano Longo

**Affiliations:** 1 Department of Biomedical Sciences for Health, Università degli Studi di Milano, 20129 Milan, Italy; marco.duca@unimi.it (M.D.); giampietro.alberti@unimi.it (G.A.); marcello.iaia@unimi.it (F.M.I.); stefano.longo@unimi.it (S.L.); 2 Department of Biotechnology and Life Sciences (DBSV), University of Insubria, 21100 Varese, Italy; damiano.formenti@uninsubria.it

**Keywords:** testing, team sport, strength training, plyometrics, youth

## Abstract

This study aimed to investigate the effects of a five-week compound training (with strength and plyometric exercises performed on separate days) on sprint, change of direction, and vertical jump in young soccer players. Eighteen novices in strength and plyometric training were assigned to either a compound training (CMPT) or a control condition (CNT). Both groups trained three times per week. One session was dedicated to soccer-specific drills. The other two weekly sessions were dedicated to circuit-based training routines employing on one-day strength exercises and on the other day plyometric exercises in the CMPT group. At the same time, the CNT group performed two weekly soccer-specific training sessions. All players were tested by 15-m sprint, change-of-direction and acceleration test (CODAT), squat jump, and countermovement jump with arms swing tests. CMPT group improved CODAT, squat jump and countermovement jump to a higher extent compared to CNT group (large vs small or trivial effects, *p* < 0.05), while both groups had similar 15-m sprint performance (*p* > 0.05). These results support the use of compound training to improve change of direction and vertical jump performances in young novice soccer players, which are unfamiliar with structured and advanced strength and plyometric training.

## 1. Introduction

The importance of performing power and strength-related tasks along with a regular sport-specific routine is well documented in youth soccer [1,2,3,4,5,6,7]. Besides the soccer-specific stimuli, practitioners are constantly prompted to employ different training programs aimed to develop players’ strength and power, providing them with beneficial effects on the pitch in terms of jump, sprint and change of direction [6].

Compound training is an alternative to well-established methods (e.g., complex training) [4,8], in which strength and plyometric exercises are performed on separate days [8,9,10,11]. As an example, the program can be structured with players training strength on the first day by executing exercises at relatively low contraction speed (e.g., loaded squat exercise), and training plyometrics after two days by performing exercises at relatively high contraction speed (e.g., unloaded drop jump or squat jump). Some studies [9,10,11] reported that this temporal exercise distribution can be effective to improve muscle power and physical performance in healthy team sport athletes. Indeed, it seems that compound training influences the force-velocity curve by promoting higher increases in lower-body strength and power compared to traditional methods (e.g., higher- or lower-load strength training) [12,13]. Combining strength and plyometric exercises seems to take the advantage of training different movement speeds and contraction times, which, in turn, lead to an enhancement of the players’ stretch-shortening cycle ability [5,8]. Therefore, key sport-specific movements (e.g., change of direction and jumps) utilising the stretch-shortening cycle would benefit from compound training. 

Interestingly, it has been reported that young athletes seem to be highly responsive to the benefits of compound training [14,15,16] probably because of the synergistic action of both the training-induced effects on performance and the growth and maturation processes involving an increasing neural regulation. This is also shown in both pre-peak height velocity and post-peak height velocity young players [15,16]. However, maturing players may potentially experience a phase of “motor awkwardness”, during which a motor control alteration may occur together with an increased susceptibility to injury. In this context, the distribution of strength and plyometric training on diverse days can be an important factor to take into account the training load and recovery handling in young players [17], particularly in those with little strength and plyometric training experience. Indeed, plyometric training may induce delayed onset of muscle soreness, acute sprains, and acute strains [5], and, consequently, it should be incorporated with caution during a weekly routine in a young population [17,18]. Moreover, adding plyometrics or performing strength and plyometric exercises concurrently on a given day can increase the training volume in a short amount of time. In this context, exploiting the combination of strength and plyometric exercises on separate days along with the soccer-specific routine could be a suitable approach to improve physical performance (sprints, changes of direction speed and jumps).

Despite the reports in the literature on the effects of compound training on physical performance in adult team sport athletes [10,11], to the best of our knowledge no information is available in young soccer players, especially in those unaccustomed to structured and advanced strength and plyometric training. In soccer training, the integration of compound training versus regular soccer-specific regimens itself might be an alternative strategy to improve players’ sprints, changes of direction speed and jump performance.

Therefore, the aim of this study was to investigate the effects of a compound training program against a sport-specific training program on sprint, change of direction speed, and vertical jump in young novice soccer players. We hypothesised that compound training would enhance players’ physical performance in terms of sprint, change of direction and vertical jump. 

## 2. Materials and Methods

### 2.1. Subjects

Eighteen sub-elite under 16 (males) soccer players voluntarily participated in the study and were randomly assigned to either an experimental (n = 9; age 14.2 ± 0.4 years; height 1.70 ± 0.08 m, body mass 56.6 ± 3.3 kg; maturity offset 1.09 ± 0.32 years) or a control group (n = 9; age 14.2 ± 0.3 years, 1.69 ± 0.07 m, body mass 55.3 ± 3.7 kg, maturity offset 1.02 ± 0.36 years). All players were accustomed to regular sport-specific training exposure as part of their weekly routine including three training sessions (lasting ~2 h) and a match-day per week. Accordingly, inclusion criteria comprised adequate training status (at least 6–8 h of training and 1 match per week), training background (a minimum of 7 years of soccer training background), unfamiliarity with structured and advanced strength and plyometric training, and absence of lower-limb injuries within the last year before the study. The players, parents or legal guardians were deeply informed about the purpose and potential experimental risks of the research before given their written informed consent to participate. The Università degli Studi di Milano granted ethical approval to carry out the study (approval number: 2/12) in accordance with the declaration of Helsinki.

### 2.2. Procedures

A randomised pre-post parallel group design was employed to investigate the effects of a short-term (i.e., 5 weeks) compound training program on sprint, change of direction and vertical jump performance in young soccer players. The duration of the training intervention is supported by a previous meta-analytical review in which performance improvements from combining strength and plyometric training can be found within a minimum of 4 weeks [2]. 

Since players were unfamiliar with structured strength and plyometric training programs, a 4-week familiarization period (2 sessions per week) was provided to all players to get them accustomed to proper exercise technique (24) before the first testing session. Then, the participants were randomly allocated to either a compound training group (CMPT) or a control group (CNT) on the last day of the familiarization period. 

Testing sessions before and after the training program included sprint, change of direction and vertical jump assessments, and were randomly administered on an artificial turf at the same time of the day (between 5.00 p.m. and 7.00 pm). All participants were asked to refrain from ergogenic drinks 24 h prior to the test day. A standardised 10-min warm-up with running drills (e.g., forward and backward running) and dynamic stretching was allowed before each testing session [7]. In the pre-test session, anthropometric characteristics (height, sitting height, and body mass) were recorded, and the corresponding maturity offset was also calculated by the equation of Mirwald et al. [19].

### 2.3. Experimental Protocol

The study was conducted in-season from September to October, within the habitual soccer training routine based on 3 weekly sessions (lasting ~2 h) and match play per week. The experimental protocol lasted 5 weeks, in which the CMPT group underwent 2 sessions per week of compound training, whereas the CNT group underwent 2 sessions per week (e.g., on Tuesday and Thursday) of soccer-specific training (10 sessions in total for each group). Both CMPT and CNT performed an identical third weekly session (e.g., on Friday) based on soccer-related drills [20] and a soccer match at the end of the week (48 h from the last training session). Of note, the two sessions per week of both interventions (CMPT and CNT) were arranged at the same time of day and matched for the same amount of time. Moreover, to achieve a high ecological validity, the beginning and the end of each session consisted of the same soccer-related drills of the third weekly session (~15 min of technical drills and ~20 min big-sided games). 

Compound training protocol. The exercises included in the protocol were completed at the beginning of two separate soccer training sessions, with at least of 48 h in between. In Session 1, the protocol included multi-joint resistance exercises to allow different multi-planar movements across the strength-velocity continuum and underpinning greater stimuli of balance and stability [21]. The players were grouped in trios and exercised in a 3-station circuit, in which strength-related exercises with low-moderate external loads were paired with core stability exercises. In Station One, while one player performed barbell front squats for 15 repetitions, another player held plank position, and the remaining player rested. In Station Two, while one player executed a barbell hip thrust for 15 repetitions, another player, while standing on one leg, hinged forward at the hip until the torso got parallel to the ground and reached backward with the opposite leg, and the third player rested. In Station Three, while one player completed 15 alternating side lunges with dumbbells, another player held a side plank position, the third player rested. Players performed the exercises in each station for three rounds, before moving to the next station. Participants spent ~9 min in each station, with a work-to-rest ratio of 1:2 or about 2 min rest before repeating the same exercise. An additional minute was conceded for changing station. The circuit-based approach was selected to better diversify and dynamise the training experience of each participant and not to undermine the perceived enjoyment of the strength-related exercises [12]. During the 5-week program, training intensity progressively increased for barbell front squat, barbell hip thrust, and alternating side lunges exercises by modulating exercise effort. The progressive overload was selected via the repetition in reserve (RIR) method [22], where the RIR value indicated the number of repetitions (reps) each participant could still perform at the end of a set based on his perceptual assessment; the selected RIR values were: 5 reps at Week 1; 4 reps at Weeks 2 and 3; and 3 reps at Weeks 4 and 5. Being unaccustomed to heavy strength training, the low relative intensity guaranteed that the players executed each repetition with a sound form. Session 1 lasted ~30 min, and its content is detailed in Table 1. Two experienced and qualified instructors supervised the CMPT group to ensure that participants performed each exercise with the proper technique and to provide verbal encouragement.

Session 2 lasted ~15 min; the players performed two horizontal jump-related drills: #1, from a double leg stance, 5 subsequent double legged hurdle jumps over five obstacles (15 cm height) and a final 5 m sprint; #2, from a single leg stance, 5 diagonal bounds into circles and a final 5 m sprint. After each set, players were allowed 45 s of passive recovery and an additional 2 min rest period was conceded between drills. The number of sets performed for each drill progressively increased every other week (4 sets at Week 1; 5 sets at Weeks 2 and 3; and 6 sets at Weeks 4 and 5). Hence, training volume was modulated by the number of jumping contacts [2]. Additionally, jumping intensity was modified according to the plyometric training guidelines for young soccer players [2] by increasing the distances between obstacles in drill #1 (1.00 m at Weeks 1 and 2; 1.50 m at Weeks 3 and 4; and 2.00 m at Week 5) and between circles in drill #2 (1.50 m at Weeks 1 and 2; 1.75 m at Weeks 3 and 4; and 2.00 m at Week 5).

In the CNT, participants underwent two sessions based on long (~30 min) and short (~15 min) intensive sport-specific stimuli. In the first session, the players performed 4 × 5 min 4-a-side games (20 × 30 m sized + 1 wildcard, work-to-rest ratio = 1:2). In the second session, the players performed 5 × 30 s of 2 versus 1 and 1 versus 2 evasion drills with a combination of defensive and offensive manoeuvres (with a constant work-to-rest ratio = 1:2). An additional 2-min rest period was conceded between the two drills. Regarding the third training session, both CMPT and CNT groups continued their weekly soccer training routine mainly consisting of: (i) ~15 min of technical drills (combination of ball retention, passing, crossing, kicking and heading); (ii) ~20 min of tactical drills (attacking/defending manoeuvres and offensive/defensive set plays); (iii) ~20 min of 4-a-sided small-sided games (16 m × 24 m sized + 1 wildcard, 1/2/3 touches,); and (iv) ~20 min of 4-a-sided big-sided games (24 m × 36 m sized + 1 wildcard, 1/2/3 touches,) with no additional strength and plyometric exercises. During each training session, a qualified strength and conditioning coach flanked by a soccer coach supervised the CNT throughout the experimental period.

### 2.4. Sprint Performance

The participants performed three trials of 15 m sprint starting from a standing position with a free departure. The foremost foot was placed 0.3 m behind the starting line. The trials were separated by 3 min of passive rest. In case the subjects slipped prior to starting, his trial was nullified, and another attempt was allowed after a recovery of 2 min. Sprint times were recorded by electronic timing gates (Witty, Microgate, Bolzano, Italy), fixed at a height of 0.7 m. The best performance was considered for the analysis. 

### 2.5. Change of Direction Speed Assessment

To assess change of direction, the change-of-direction and acceleration test (CODAT) was used. The CODAT involves a straight 5 m sprint, followed by three 3-m sprints including four change of directions at 45°, 90°, 90°, and 45°, respectively [23], performed with a side-step technique. Then, a further straight 10 m sprint completed the course. Sprint times were recorded by electronic timing gates, fixed at a height of 0.7 m. Three trials with 3 min of rest in-between were executed, and the best trial was considered in the analysis. According to the protocol adopted by Lockie et al. [23], subjects rocking backwards or hesitating prior to starting were allowed to recover and repeat the trial after 2 min.

### 2.6. Vertical Jump Assessment

Squat jump. While maintaining the arms in akimbo stance, the players were asked to maintain a squatting position at ~90° of knee flexion for 3 s, and then to jump vertically. A video-based observation was used to evaluate the proper execution. Jump flight time was recorded by a photoelectric system (Ergojump, Bosco System, Roma, Italy), and the corresponding jump height was computed. Each subject performed three trials interspersed by 2 min of passive recovery. In case of an incorrect execution, the subject repeated the trial after an additional 1-min recovery. The best jump height was considered in the analysis. 

Countermovement jump with arm swing. The participants performed three jumps with arm swing, each one separated by 2 min of rest. In case of incorrect execution, the subject repeated the trial after an extra 1-min recovery. An experienced and qualified instructor supervised the subjects’ adherence to the provided jumping instructions. The supervisor also ensured that, during the flight phase, the lower limbs were kept extended, thus avoiding an increase in flight time and jump height.

Jump flight time was recorded by a photoelectric system (Optojump Next System, Microgate, Bolzano, Italy), and the corresponding jump height was computed. The best jump height was considered in the analysis. 

### 2.7. Statistical Analysis

The Shapiro-Wilk’s test was conducted to verify data distribution. Test-retest reliability was performed using the intraclass correlation coefficient (ICC) for sprint, change of direction, and vertical jump tests. The unpaired student’s *t*-test was used to detect possible differences in all performance and anthropometric variables at the pre-test session between groups. A two-way mixed-model analysis of variance (ANOVA) was used to detect interactions (time x intervention) and main effects of time (within-group changes) and group between CMPT and CNT throughout the 5-week experimental protocol. Significance was set with *p* ≤ 0.05. The analysis was performed using the IBM SPSS® Statistics software (v. 21, New York, NY, USA). The Hedge’s *g* effect size [24] of each performance change was calculated, and threshold values were g < 0.2 (trivial), 0.2 < g < 0.5 (small), 0.5 < g < 0.8 (moderate), and g > 0.8 (large). Data are presented as mean ± SD. Relative changes were expressed as means ± 95% confidence intervals (CI).

## 3. Results

Average ICC values ranged from good to excellent values (15-m sprint: ICC = 0.96, 95% CI = 0.86 to 0.99; CODAT: ICC = 0.89, 95% CI = 0.61 to 0.98; squat jump: ICC = 0.92, 95% CI = 0.71 to 0.98; countermovement jump with arm swing: ICC = 0.93, 95% CI = 0.73 to 0.98). The two groups did not differ in height (*p* = 0.92) and body mass (*p* = 0.84). In the pre-test values, no significant differences were found in 15-m sprint (*p* = 0.67), CODAT (*p* = 0.99), squat jump (*p* = 0.35) and countermovement jump with arm swing (*p* = 0.78) between CMPT and CNT.

**Figure 1:**
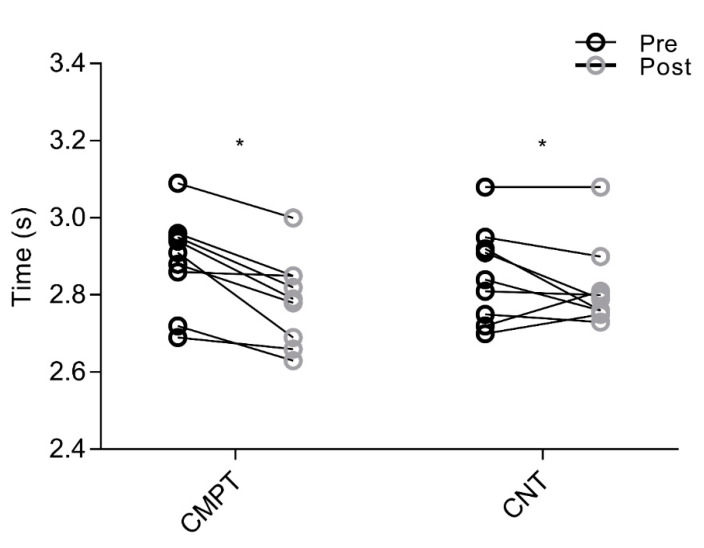
Individual changes in 15 m sprint performance between CMPT and control condition (CNT). The circles with black outline show the mean pre-values while the circles with grey outline indicate the mean post-values for both interventions. * = significant (*p* ≤ 0.05) within-group (pre and post) changes. Note: CMPT = compound training group, CNT = soccer-specific training group.

**Figure 2:**
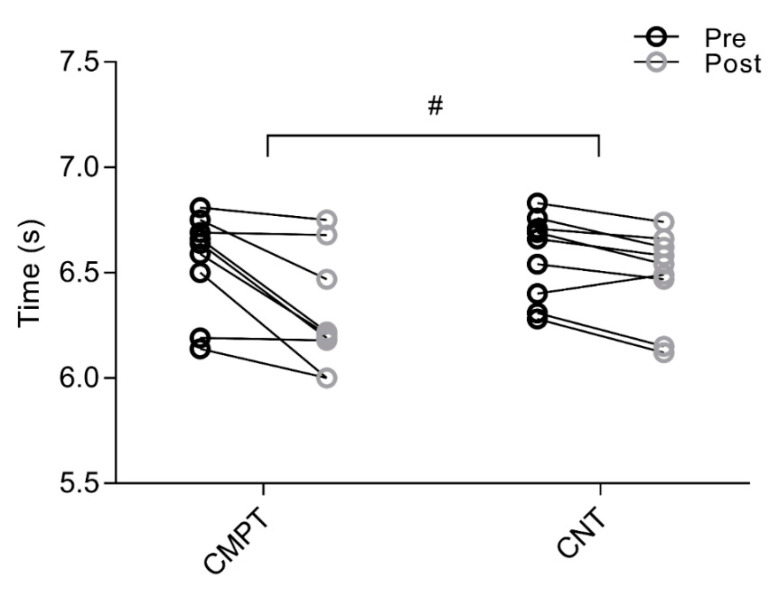
Individual changes in change of direction performance between CMPT and CNT tested by change-of-direction and acceleration test (CODAT). The circles with black outline show the mean pre-values while the circles with grey outline indicate the mean post-values for both interventions. # = significant (*p* ≤ 0.05) interaction. Note: change of direction= change of direction speed, CMPT = compound training group, CNT = soccer-specific training group, CODAT= change of direction and acceleration test.

**Figure 3:**
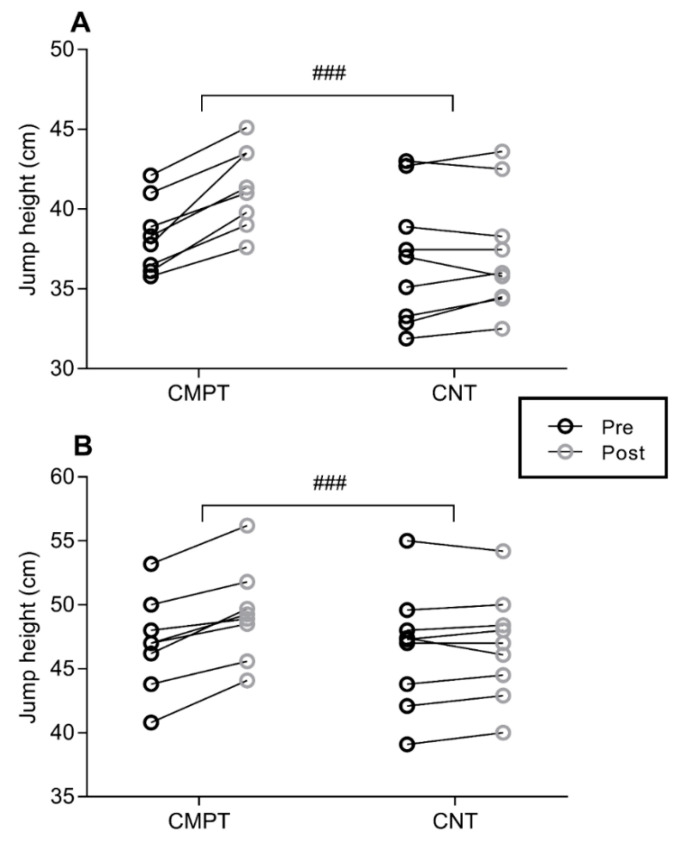
Individual changes in vertical jump performance between CMPT and CNT. Panel A and B show squat jump and countermovement jump with arm swing outcomes, respectively. The circles with black outline show the mean pre-values while the circles with grey outline indicate the mean post-values for both interventions. ### = significant (*p* ≤ 0.001) interaction. Note: CMPT = compound training group, CNT = soccer-specific training group.

For 15 m sprint, no interaction was revealed (F_(1,16)_ = 0.435, *p* = 0.053). A main effect of time was found (*p* < 0.001), while there was no main effect of group (*p* = 0.82) (Figure 1). Pre-post changes showed that sprint time improved from 2.89 ± 0.12 s to 2.79 ± 0.11 s with large (g = 0.82, 95% CI = −0.16 to 1.89) effect in CMPT, and from 2.85 ± 0.12 s to 2.82 ± 0.11 s with small (g = 0.24, 95% CI = −0.72 to 1.24) effect in CNT. For CODAT, an interaction was detected (F_(1,16)_ = 5.119, *p* = 0.038) (Figure 2). A main effect of time was also found (*p* < 0.001), while there was no main effect of group (*p* = 0.33). Pre-post changes showed that CODAT enhanced from 6.55 ± 0.24 s to 6.30 ± 0.27 s with a large (g = 0.93, 95% CI = −0.06 to 2.02) effect in CMPT, and from 6.58 ± 0.20 s to 6.49 ± 0.22 s with small (g = 0.40, 95% CI = −0.56 to 1.42) effect in CNT, respectively. Regarding vertical jump, interactions were detected for both squat jump (F_(1,16)_ = 27.261, *p* < 0.001) and countermovement jump with arm swing (F_(1,16)_ = 21.151, *p* < 0.001) (Figure 3). Main effects of time (*p* < 0.001) were detected, while there were no main effects of group for both vertical jump tests (*p* = 0.08 and *p* = 0.43, respectively). In CMPT, squat jump increased from 38.38 ± 2.15 cm to 41.32 ± 2.39 cm with a large effect (g = 1.23, 95% CI = 0.20 to 2.38), whereas countermovement jump with arm swing increased from 47.11 ± 3.51 cm to 49.22 ± 3.46 cm with moderate effect (g = 0.57, 95% CI = −0.39 to 1.61). In CNT, squat jump and countermovement jump with arm swing changed from 36.92 ± 4.07 cm to 37.23 ± 3.72 cm, and from 46.59 ± 4.57 cm to 46.79 ± 4.13 cm, respectively, with trivial effects (g = 0.07, 95% CI = 0.90 to 1.05, and g = 0.04, 95% CI = −0.93 to 1.02, respectively).

## 4. Discussion

The present study aimed to investigate the effects of a 5-week compound training program on sprint, change of direction, and vertical jump performance in young novice soccer players. The main finding indicates that CMPT improved CODAT, squat jump and countermovement jump with arm swing to a higher extent than CNT. These results support our hypothesis that young players would benefit from a short-term compound training program, which enhanced their change of direction and jumping abilities.

Young soccer players should develop physical abilities optimally to cope with the increasing match and training demands throughout the season. Moreover, due to the continuous changing context during a match, young players need to become more effective in applying force within a brief amount of time in several activities involving a stretch-shortening cycle (acceleration, deceleration, change of direction, and jump tasks) in a multidirectional manner [4,7,25,26]. This scenario requires trainers to identify effective training programs for enhancing strength and power transferable to youth sport-specific performance [8], such as compound training. Moreover, the peculiarity of compound training (combining strength and plyometric training separately) might limit the increase of training volume in a short time that, without an adequate recovery, could decrease the players’ performance [17].

Previous soccer-related studies focused on the effects of strength and plyometric training performed within the same session (i.e., complex training) reporting consistent improvements in sprint [27] and jump performance [12] in both novice and expert players. However, to the extent of our knowledge the present study is the first investigating the effects of a training program derived from the separation of strength and plyometric exercises in two non-consecutive days on physical performance in young novice soccer players. The current changes in CODAT demonstrated that compound training can provide transferable effects on change of direction tasks, likely depending on an improvement of the stretch-shortening cycle ability, compared with a soccer-specific training regimen alone. For instance, during a change of direction, players have to manage the forces applied to the ground in the appropriate direction to enable a quick switch from the eccentric (deceleration phase) to the concentric (acceleration phase) knee extensors muscle action [28]. This rapid switch between the two phases has been well-documented to be involved in plyometric training, which increases muscular power and movement efficiency expressed during a change of direction [28] via an enhanced inter- and intramuscular motor unit recruitment and stretch reflex responses [5]. Hence, the greater improvement in CODAT performance by CMPT compared to CNT may be largely attributable to the neuromuscular adaptations derived from both strength and plyometric training (e.g., double legged hurdle jumps and diagonal bounds).

In the current study, squat jump and countermovement jump with arm swing remarkably improved in CMPT (~7.5% and ~4.5%, respectively) compared to CNT (~1.4% and ~1.2%, respectively). This is in line with the study of Mihalik et al. [10] that employed a short-term compound training program for team sport athletes. The authors reported that both male and female volleyball players improved considerably their countermovement jump with arm swing performance after four weeks of compound training. These results may be likely attributable to neuromuscular changes (e.g., motor unit recruitment), which have been shown to be the primary adaptation to both strength and plyometric training in youth [5]. Accordingly, squat jump can inform about the ability to briefly develop force in concentric muscle actions, while countermovement jump with arm swing can inform about the ability to briefly produce force in movements involving a stretch-shortening cycle, switching rapidly from eccentric to concentric muscle actions. In this context, the compound training may have improved motor unit recruitment during concentric dynamic (e.g., squat jump) and stretch-shortening cycle movements (change of direction and countermovement jump with arm swing).

Regarding acceleration, both CMPT and CNT showed similar improvements in 15-m sprint after five weeks of training. This result seems partially in line with another study applying a six-week complex training program in under 18 soccer players [27]. Although combining strength and plyometric exercises within the same training day improved 5- and 15-m sprint times, they did not show concurrent changes in the control group. One explanation for this discrepancy could be due to the present soccer-specific training performed by CNT. Indeed, during small- and- big-sided games accounting for ~45 % of the total session time, players continuously engaged in several sprints over short distances to address the sport-specific demands (e.g., evading or marking fast an opponent, winning duels effectively, and gaining the ball possession quickly). Therefore, it is reasonable to assume that the repeated sprint-based activities experienced by CNT may have provided a sprint stimulus, thus explaining the similarity between groups and the discrepancy with the literature. However, Maio Alves et al. [27] did not clarify the specific contents of their soccer training routine and any further comparative discussion is limited. In contrast to the present results, Cavaco et al. [29] did not observe improvements in 15-m sprint after a six-week complex training in young players. The authors explained their results with the interindividual variability in running technique according to the maturation age. However, neither kinematic analysis nor maturity assessment were performed [29]; thus, such an explanation should be taken with caution. Of note, Cavaco et al. [29] did not clearly specify whether the players were novice or expert in strength and/or plyometric training. Indeed, training adaptation can differ upon players’ exercise background [30] and it might likely explain the present 15-m sprint improvements of both CMPT and CNT, as well as the contrasting findings in respect to Cavaco et al [29]. 

The current study presents three main limitations that should be acknowledged. First, we did not directly control for maturity status, but only indirectly by the maturity offset calculation, which were averagely homogeneous between groups and not included within the analysis. Nevertheless, the maturity offset goes with a standard error [19] and might limit the interpretation when only the average is considered. Second, change of direction performance was measured by total time, which has been observed being mainly influenced by linear sprint ability. As such, future studies isolating accurately the different components of change of direction performance are desirable [31]. Third, even though the present sample size was in line with previous studies in team sport athletes [11,29], its small size limits the interpretation of the results as well as their generalization. Further studies with larger sample are warranted. 

In summary, the change of direction and vertical jump performance of young soccer players with no strength training experience can be improved by a short-term compound training programme based on strength and plyometric exercises performed on two separate days. Importantly, changing direction rapidly in one-to-one situations or jumping high during aerial duels is pivotal to successfully compete in soccer. Therefore, choosing a training strategy exploiting strength and plyometric exercises (i.e., compound training) to enhance players’ performance is desirable. In addition, practitioners dealing with young novice individuals might be encouraged to employ this approach within their training design instead of focusing exclusively on soccer-specific stimuli. It is worth noticing that the present approach was tailored to cope with the training youth guidelines, considering appropriate technical execution, exercise selection, training intensity and volume, and training experience [12]. However, young players with various levels of play (i.e., elite and sub-elite) would likely benefit from different exercise intensity and volume.

## 5. Conclusions

The present findings showed that a relatively short-term compound training program can enhance change of direction and vertical jump performance in young soccer players. Compound training may be integrated as an alternative training strategy for practitioners dealing with novices in structured strength and plyometric training. Future studies are warranted to investigate the effects of compound training with different volume and intensity in the attempt to establish an optimal dose-response relationship between training load, performance enhancement, and recovery time in young soccer players.

## Figures and Tables

**Table 1:** A detailed description of the training protocol performed by compound training group (CMPT) during Session 1.

Weekly Experimental Protocol						
Station	Exercise	Week 1	Week 2	Week 3	Week 4	Week 5
ONE	a. Barbell Front Squat	3 S × 15 R @ RIR 6	3 S × 15 R @ RIR 5	3 S × 15 R @ RIR 5	3 S × 15 R @ RIR 4	3 S × 15 R @ RIR 4
	b. Plank	Isometric 30 s	Isometric with alternating forward arm reach 30 s		Isometric with alternating forward arm and upward leg reach 30 s	
TWO	a. Barbell Hip Thrust	3 S × 15 R @ RIR 6	3 S × 15 R @ RIR 5	3 S × 15 R @ RIR 5	3 S × 15 R @ RIR 4	3 S × 15 R @ RIR 4
	b. Standing Superman	Isometric 30 s e.s.	Isometric with contralateral forward arm reach 30 s e.s.		Isometric with forward arms reach 30 s e.s.	
THREE	a. Alternating Side Lunges with dumbbells	3 S × 15 R @ RIR 6	3 S × 15 R @ RIR 5	3 S × 15 R @ RIR 5	3 S × 15 R @ RIR 4	3 S × 15 R @ RIR 4
	b. Side Plank	Isometric 30 s e.s.	Isometric with free arm abduction in the frontal plane 30 s e.s.		Isometric with free arm and leg abduction in the frontal plane 30 s e.s.	

Note: CMPT = compound training group; BW = body weight, e.s. = each side, S = sets, R = reps, RIR = repetitions in reserve.
